# Rising temperatures favour defence-suppressing herbivores

**DOI:** 10.1007/s10340-024-01781-2

**Published:** 2024-04-20

**Authors:** Jéssica Teodoro-Paulo, Jacques A. Deere, João Valeriano-Santos, Steven Charlesworth, Alison B. Duncan, Merijn R. Kant, Juan M. Alba

**Affiliations:** 1 Centre for Ecology, Evolution and Environmental Changes and Global Change and Sustainability Institute (cE3c - CHANGE), Faculty of Sciences, University of Lisbon, Lisbon, Portugal; 2 Institute for Biodiversity and Ecosystem Dynamics, University of Amsterdam, Amsterdam, The Netherlands; 3 Institut des Sciences de l’Évolution, University of Montpellier, Centre National de la Recherche Scientifique (CNRS), Institut de Recherche pour le Développement-Motpellier (IRD-Montpellier), Montpellier, France; 4 Institute for Mediterranean and Subtropical Horticulture La Mayora, Universidad de Malaga-Consejo Superior de Investigaciones Científicas (IHSM-UMA-CSIC), Málaga, Spain

**Keywords:** Climate change, Heatwave, *Tetranychus*, Spider mites, Plant–herbivore interactions

## Abstract

Rising temperatures due to climate change are predicted to accelerate the life cycle of arthropod herbivores thereby exacerbating pest formation. Notorious pests like spider mites thrive in areas with high temperatures (32–35 °C), and it is predicted that the size and number of such areas will expand in the coming decades. Higher temperatures can directly accelerate population growth, but also indirectly affect them through changes in the plant's defensive mechanisms. Spider mites have been shown to adapt to plant defences, with natural selection favouring defence-suppressing traits. However, it is not known to what extent suppression is affected by rising temperatures and how this might tie into the rate of adaptation and pest damage. In this study, we investigated the effect of two temperatures (25 °C and 32 °C), on the spider mite–tomato interaction, predicting the influence of rising temperatures on favouring defence-adapted mites. We found that all mite strains caused more plant damage at 32 °C, but temperature did not affect the overall patterns of induction and suppression of defence genes. Although fecundity was higher for all strains at 32 °C, juvenile and adult survival was lower, especially for inducer mites. With these data, we parametrized population models for the two strains over three months, indicating that suppressor mites might displace inducers at the higher temperature, either when it is constant or in the form of heat waves. Our models predict that in areas with higher temperatures, defence-suppressing mites are favoured, which will accelerate and consequently spur pest formation.

**Supplementary Information:**

The online version contains supplementary material available at 10.1007/s10340-024-01781-2.

## Introduction

Global warming is predicted to have a tangible impact on terrestrial organisms and their interactions (Bale et al. 2002; Logan et al. 2003; Deutsch et al. 2018) and is thought to be the largest anthropogenic disturbance in nature (Sala et al. 2000; Dunn 2005). The impact of global warming will vary geographically (Dunn 2005; Parmesan and Yohe 2003; Root et al. 2003), but an important determinant of biological responses will depend on the degree of warming itself and on the temperature tolerance of different species. Ectothermic organisms like insects and other arthropods are especially vulnerable to global warming as their basic physiological functions (i.e. locomotion, rates of development, reproduction, metabolism, and consumption) are strongly influenced by environmental temperatures (Bale et al. 2002). However, not only the physiology of ectothermic species may be influenced by rising global temperatures, but also their interactions with competitors (Porras et al. 2018), predators (Jaworski and Hilszczański 2013) and, in the case of herbivores, their host plants (Nechols et al. 2020).

Temperature can alter the ability of host plants to establish defences which can, in turn, affect herbivore performance (Dury et al. 1998). Several studies have shown that temperature can modify traits that influence plant defences, such as nutritional content, phytotoxin accumulation and other important biochemical processes, and this effect is often negative (e.g. Himanen et al. 2008; Diamond and Kingsolver 2010; Bauerfeind and Fischer 2013; Paudel et al. 2020). For instance, high temperatures can alter constitutive defences such as the composition of trichome exudate in *Geranium*, reducing viscosity and thereby resistance (Walter et al. 1991). Hormones involved in inducible defences are also affected by temperature, as shown by the increased production of jasmonic (JA) and salicylic (SA) acid at high temperatures (Clarke et al. 2009; Maimbo et al. 2007). However, how elevated temperatures affect plant resistance to herbivores will vary across different plant–herbivore systems (reviewed in Nechols et al. 2020).

Higher temperatures have been associated with an increase in reproduction, growth and voracity as well as expansion of the host range by herbivores and arthropod pests (Zvereva and Kozlov 2006; Deutsch et al. 2018). Furthermore, temperature can have a strong effect on their reproduction, albeit differently for different species. For instance, for some species oviposition increases (e.g. aphids; Ma et al. 2015), for others it reduces (e.g. parasitic wasps, Zhang et al. 2019) or even stops completely (e.g. fruit flies, Evans et al. 2018) when at temperatures higher than their optimum. In addition, Paudel et al. (2020) found that the effect of elevated temperatures on plant–herbivore interactions can be asymmetric, i.e. it can increase herbivore growth rate and at the same time reduce the growth rate of the plant and its ability to establish defences, thus exacerbating pest growth. The effects of temperature on plants and herbivores are often investigated in the scope of climate change (reviewed in Nechols et al. 2020). This is especially relevant since herbivores may exacerbate the reduction in productivity of major crop plants due to rising temperatures and more readily become pests (Zhao et al. 2017).

Inducible plant defences are shaped by a complex network of biochemical pathways (War et al. 2012), which are initiated after the herbivore has been detected (Wu and Baldwin 2009). However, some herbivores can interfere with the appropriate execution of these defences for example by suppressing them (e.g. Musser et al. 2002; Alba et al. 2015). Suppression of defences is often attributed to salivary effectors secreted into the plant during feeding (Musser et al. 2002; Lawrence et al. 2008; Naessens et al. 2015), which interferes with the formation of defensive end products (Zhu-Salzman et al. 2005), thereby improving the host as food and, consequently, its susceptibility to the attacker. Temperature was found to correlate with the effectiveness of defence suppression since the activity of the GOX salivary effector in the caterpillar, *Helicoverpa zea*, is lower at 35 °C compared to 25 °C (Paudel et al. 2020). This suggests that temperature may not only influence herbivore life history but also its capacity to interact with the defences of its host. However, little is known about how such effects may play out at the level of herbivore population growth.

Some species of herbivorous spider mites (Tetranychidae) are well known for their ability to manipulate JA and SA defence pathways in tomato plants (*Solanum lycopersicum*) to boost their performance (i.e. reproduction and population growth; Kant et al. 2008; Sarmento et al. 2011; Alba et al. 2015; Godinho et al. 2016; Paulo et al. 2018). The two-spotted spider mite *T. urticae* is a highly polyphagous species, which has been found to feed on over 1100 plant species (Dermauw et al. 2013). The red spider mite *T. evansi* is found mostly on nightshades and is an invasive pest on solanaceous crops (predominantly tomato) throughout Southern Europe and Africa (Boubou et al. 2012). Spider mites are major agricultural pests and have been reported to feed from numerous crops such as tomato. However, their control (e.g. via biological control or pesticides) is notoriously troublesome (Navajas et al. 2013; Agut et al. 2018). Defence suppression by these mites is associated with salivary proteins referred to as effectors (Villarroel et al. 2016; Jonckheere et al. 2018). Effector profile expression is fine tuned depending on different factors, like plant host (Jonckheere et al. 2006, 2018); developmental stage and sexes (Liu et al. 2020b); the presence of competitors (Schimmel et al. 2017); and on the light/dark cycle (Liu et al. 2020a).

In this study, we assessed how different temperatures affect the ability of spider mites to manipulate plant defences and how this affects their strain growth and, through population modelling, their ability to compete with spider mites that induce plant defences. Largely depending on the host plant identity, relative humidity and mite age, increasing temperatures up to about 35 °C (*T. urticae,* Bayu et al. 2017) or 40 °C (*T. evansi*, Gotoh et al. 2010) decreases their developmental time. For this study, we used three spider mite strains: two that suppress tomato defences (i.e. the tomato specialist *T. evansi* and a tomato-adapted strain of *T. urticae*) and one strain of *T. urticae* that does not (a bean-adapted strain of *T. urticae* that induces tomato defences). We subjected tomato plants and herbivorous mites to different temperature regimens (25 °C and 32 °C) and evaluated the effect of these temperatures on plant defences and mite performance. Subsequently, we used these data to parameterise a population dynamic model. The impact of temperature on the population dynamics of inducer and suppressor herbivores and the consequences on pest formation and adaptation is discussed.

## Material and methods

### Plants

Tomato plants (*Solanum lycopersicum* L.) of the varieties Castlemart (for the experiment and mite maintenance) and Moneymaker (for mite maintenance), along with bean plants (*Phaseolus vulgaris* L. cv Speedy) used for maintenance and cohorts of mites, were sown in a glasshouse (photoperiod 16: 8 h, 25: 18 °C, day: night, 60% relative humidity (RH)) for 21 and ten days, respectively. Tomato plants (cv Castlemart) used in the experiments were germinated in the glasshouse, and then, at ten days old, transplanted to 15ø cm pots and transferred to a climate chamber (photoperiod of 16 h light:8 h dark, 25 °C, day:night, 50–60% RH) and allowed to grow until 28 days of age. One day before mite infestation, plants were transferred to the experimental climate chambers set at 25 °C and 32 °C (photoperiod of 16: 8 h, 60% RH) for acclimatization.

### Spider mites

In this study, two mite species were used, *T. urticae* and *T. evansi*. We used the *T. urticae* Santpoort-2 strain that induces defences in tomato (Alba et al. 2015), a *T. urticae* Outbred strain that suppresses tomato defences (Godinho et al. 2020) and *T. evansi* Viçosa-1 strain that suppresses defences (Alba et al. 2015). The *T. urticae* inducer strain had been maintained on detached bean leaves, the *T. urticae* suppressor strain on detached tomato leaves (cv Moneymaker) for four years (Godinho et al. 2020) and the *T. evansi* strain on detached tomato leaves (cv Castlemart) for at least five years (Alba et al. 2015). All strains were maintained in a climate chamber (25 °C, photoperiod of 16: 8 h, 60% RH). Fourteen days before the experiment, female cohorts of 70 individuals were created, on their respective hosts, on detached leaves. This was done to obtain 13 ± 1-day-old females that were used for plant infestation. One day before infestations, females were transferred to the experimental climate chambers (25 °C or 32 °C, photoperiod of 16: 8 h, 50–60% RH) for acclimatization.

### Exposure to 25 °C and 32 °C

Two different temperatures were selected: 25 °C as it is the regular temperature for mite maintenance and experiments (considered as the control temperature) and 32 °C as it is within the range of the optimal temperature of both *Tetranychus* species used and tomato. The optimal temperature range for *T. urticae* is between 27 and 32 °C (Kim et al. 2008), with a maximum rate of natural increase at 27 °C (Riahi et al. 2013). For *T. evansi*, the optimal temperature range is between 31 and 36 °C, with a high rate of population increase at 34 °C (Bonato 1999). During the growing season, air temperatures between 18.3 and 32.2 °C are considered suitable for tomato cultivation (Hochmuth and Hochmuth 2012). As such, both our control and high temperature fall within the optimal temperature for both mite species (*T. urticae* and *T. evansi*) and tomato growth and development.

### Spider mite infestations

Spider mites infested intact tomato plants at either 25 °C or 32 °C for two days and uninfested plants were used as control. For our treatments, we infested four leaflets per plant with 14 mated females (13 ± 1 days old) each. These were non-terminal leaflets, distributed across two leaves of each plant (see infestation scheme in Table S1). To avoid mite dispersal, the adaxial surface of the infested leaflet was isolated by placing a 1:1 mix of entomological glue (Tanglefoot, Michigan, USA) and lanolin (Sigma-Aldrich, St Louis, MO, USA) around the edges of the leaflet (see infestation scheme in Table S1). After two days of infestation, we measured several key variables as described in the sections below.

### Effect of high temperatures on the survival and oviposition of females and their plant damage

In a subset of the infested leaflets (two of the four, one from each leaf, *n* = 40 across nine temporal blocks, see Table S1 for experimental design), the number of alive and dead females and the number of eggs were counted. Assuming constant performance and mortality (Li and Zhang 2022), we then calculated the proportion of females surviving per day (1) and the fecundity per surviving female per day (2). These Eqs. (1 and 2) account for differential female mortality, which can only be assessed at the end of the assay and thus enables a more accurate representation of per capita survival and fecundity.

1 $${\text{Female }}\;{\text{survival}}\;{\text{ per}}\;{\text{ day }} = 1 - \frac{{{{{\text{dead }}\;{\text{females}}} \mathord{\left/ {\vphantom {{{\text{dead }}\;{\text{females}}} {2\;{\text{ days}}}}} \right. \kern-0pt} {2\;{\text{ days}}}}}}{{\left( {{\text{dead }}\;{\text{females}} + {\text{alive }}\;{\text{females}}} \right)}}$$

2 $${\text{Eggs }}\;{\text{per }}\;{\text{alive}}\;{\text{ female }}\;{\text{per}}\;{\text{ day }} = {{\left( {\frac{{{\text{total }}\;{\text{eggs}}}}{{{\text{alive}}\;{\text{ females }} + {{{\text{dead }}\;{\text{females}}} \mathord{\left/ {\vphantom {{{\text{dead }}\;{\text{females}}} 2}} \right. \kern-0pt} 2}{ }}}} \right)} \mathord{\left/ {\vphantom {{\left( {\frac{{{\text{total }}\;{\text{eggs}}}}{{{\text{alive}}\;{\text{ females }} + {{{\text{dead }}\;{\text{females}}} \mathord{\left/ {\vphantom {{{\text{dead }}\;{\text{females}}} 2}} \right. \kern-0pt} 2}{ }}}} \right)} {2{ }\;{\text{days}}}}} \right. \kern-0pt} {2{ }\;{\text{days}}}}{ }$$

Plant damage was assessed using pictures of the treated leaflets as follows: The same leaflets used to calculate survival and fecundity (*n* = 40, see Table S1 for experimental design) were placed between two transparent glass plates. Pictures were taken with a Nikon D750 camera with a 60 mm Nikon Macro lens. Pictures were analysed following the protocol described in Liu et al. (2020a). In brief, using Image J (Rasband 2012, http://rsb.info.nih.gov/ij/), the coloured pictures were transformed into 8-bit images and the area within the lanolin barrier was selected. Pixels were converted to mm using the average length of spider mites. Considering the length (i.e. from the tip of the abdomen to the mouth parts is 0.4 mm, Sarwar 2020). Using the threshold tool, the damaged areas were set to white, and the area was measured in mm^2^, obtaining the chlorotic leaf damage area. The damaged area was also corrected for female mortality as described below (3).

3 $${\text{Damage }}\;{\text{area}}\;{\text{ per}}\;{\text{ alive}}\;{\text{ female }} = { }\frac{{{\text{total}}\;{\text{ damage }}\;{\text{area}}}}{{{\text{alive}}\;{\text{ females }} + { }{{{\text{dead }}\;{\text{females}}} \mathord{\left/ {\vphantom {{{\text{dead }}\;{\text{females}}} 2}} \right. \kern-0pt} 2}}}$$

### Effect of high temperatures on *T. urticae* and *T. evansi* developmental time and survival

We tracked several life history characteristics during egg-to-adult development of mites under 25 °C and 32 °C on tomato leaf discs. For a subset of the leaflets used to calculate survival and fecundity (*n* = 20, see Table S1 for experimental design) we randomly isolated 20–28 eggs per leaflet. These were then isolated in groups of five to seven eggs on 15 mm Ø leaf discs on wet cotton wool at 25 °C and 32 °C. Developmental stage and survival were assessed every day and the time until the first individual reached a new developmental stage (larva, protonymph, deutonymph, male or female and quiescence stages), offspring mortality and the proportion of female offspring in each group of five to seven eggs were recorded. Also, when juveniles matured into females, the time until the first egg laid (i.e. F1 eggs) was assessed for each group. This was used as a measure for the generation time (i.e. the time from egg stage to first oviposition according to Abou-Setta and Childers 1991). This was done for all treatments across 5 temporal blocks, meaning that for each treatment life history traits of 400–560 eggs were followed. Leaf discs were taken from plants grown under the same conditions and acclimatized to each temperature for three days, as those used for the previous experiments. Note that, every 4 days, a fresh leaflet was placed next to the old one, to avoid the effect of reduced food quality and quantity on mite development and survival.

### Effect of temperature on the expression of a mite effector gene and tomato defence genes

A subset of the two remaining leaflets per plant and mites that were infesting the plant (*n* = 20, across 4 temporal blocks, see Table S1 for experimental design) were pooled and flash frozen in liquid nitrogen and stored at − 80 °C until RNA extraction. This RNA was used to produce cDNA to determine the transcript accumulation of *effector 84 (Tu84–tetur01g01000 *and *Te84–KT182961*; Villarroel et al. 2016)*.* Taking advantage of the experimental set-up, in which mites are infesting the leaflets, it was possible to extrat I RNA from both plants and mites. This provides a powerful statisitical feature by pairing the molecular data from tomato plants and mites. As such, this was achived by isolating the total RNAfrom ~ 100 mg of a ground sample using the E.Z.N.A.® Plant RNA Kit (Omega Bio-tek, Norcross, Georgia, USA). After DNAse treatment, 1000 ng/µL of RNA was converted to cDNA as described in Alba et al. (2015). 1µL of 10 times diluted cDNA was used as a template for a 20 µL quantitative reserve-transcriptase polymerase chain reaction (qRT-PCR) using the HOT FIREPol® EvaGreen® qPCR Mix Plus (ROX) (Soli Biodyne, Tartu, Estonia) and the 7500 Real-Time PCR System (Applied Biosystems, Waltham, Massachusetts, USA). Normalized gene expression (NE) was obtained using the ΔCt method (Alba et al. 2015). For graphical representation, NE was scaled per gene to the lowest mean NE. The mite *Tetranychus Ribosomal protein 49* (*RP49*) was used as a housekeeping gene for qPCR normalization. Primers and gene identifiers can be found in Villarroel et al. (2016). This cDNA was also used to determine transcript accumulation levels of tomato *Proteinase Inhibitor iIc (WIPI-iIc)* and *Proteinase Inhibitor iIf (WIPI-iIf*) as markers for JA-defences and of *Pathogenesis-related protein 1a (PR-1a*) as a marker for SA defences*. Solanum lycopersicum Actin* was used as a tomato housekeeping gene to normalize the target genes. All primers and gene identifiers for the tomato genes can be found in Alba et al. (2015).

### Statistical analysis

All statistical analyses were performed with the software R (version 4.2.2, R Development Core Team 2022, Chichester, UK).

The effect of temperature and mite strain on the per day survival of females was assessed by performing a generalized linear mixed model (GLMM) assuming a beta-binomial distribution, to account for overdispersion (glmmTMB package, Bates et al. 2015), using a cbind function between living females per day and dead females per day as the response variable. The effect of temperature and strain identity (*mite*) on feeding damage, the number of eggs per living female (i.e. fecundity), on the NE of *effector 84* and the nEs of the plant defence genes (including the clean control plants), were analysed using independent GLMMs assuming a Gamma distribution and a log effect of the fixed factors (family = Gamma(link = ”log”), lmer, lme4 package, Bates et al. 2015). Models included mite (*T. urticae* inducer, *T. urticae* suppressor or *T. evansi* suppressor), temperature (25 °C or 32 °C) and their interaction (mite*temperature) as fixed explanatory variables, and *block* as a random variable.

The time to reach each developmental stage (i.e. the time that the first individual of a new stage appeared on each leaf disc) was analysed using a GLMM assuming a Poisson distribution (glmmTMB package, Bates et al. 2015), with *mite* (*T. urticae* inducer, *T. urticae* suppressor or *T. evansi* suppressor), temperature (25 °C or 32 °C), stage (i.e. larva, protonymph, deutonymph, male or female, each quiescence stage and eggs from F1) and their interaction (mite*temperature*stage) as fixed explanatory variables, and block and leaf disc as random variables.

To analyse offspring survival during development from egg to adult on the leaf discs, Cox’s proportional hazard mixed-effect models (coxme package, Therneau 2020) were performed independently for each temperature to account for differences in generation times (from egg to reproductive adult). Accidental deaths (i.e. drowned individuals), missing individuals and individuals still alive at the end of the experiment were considered censored data. Both models included mite (*T. urticae* inducer, *T. urticae* suppressor or *T. evansi* suppressor) as a fixed explanatory variable and block and leaf disc as random variables.

To assess the effect of temperature and mite strain identity (mite) on the proportion of female offspring a GLMM assuming a binomial distribution (lmer, lme4 package, Bates et al. 2015) was used, using a cbind function between number of females and number of males as the response variable. The model included *mite* (*T. urticae* inducer, *T. urticae* suppressor or *T. evansi* suppressor), *temperature* (25 °C or 32 °C) and their interaction (mite*temperature) as fixed explanatory variables, and block and leaf disc as random variables.

For all analyses, when significant differences were found, multiple comparisons were performed using estimated marginal means (emmeans, emmeans package) (Lenth et al. 2022) and the *P*-values corrected using the false discovery rate (FDR) method (*α* = 0.05) (Benjamini and Hochberg 1995).

### Dynamics of inducer and suppressor *T. urticae* strains at a constant temperature or when exposed to heat waves

#### Two-phenotype model: inducer versus suppressor strains of *T. urticae*

We use a two-phenotype model to investigate the dynamics of an inducer strain and a suppressor strain of *T. urticae* under different temperature regimes. Within the model, inducer (NI) and suppressor (NS) strains consist of two different life stages, juveniles (NI_1_, NS_1_) and adults (NI_2_, NS_2_), with the stage-structured dynamics of the inducer (NI) and suppressor mites (NS) described by the following differential equations:

4 $$\frac{{{\text{dNI}}}_{1}}{{\text{d}}t}= {({\text{RIb}}){\text{NI}}}_{2}-\mathrm{ vI}{{\text{NI}}}_{1}$$

5 $$\frac{{{\text{dNI}}}_{2}}{{\text{d}}t}={{\text{eINI}}}_{1}- \mathrm{\mu I}{{\text{NI}}}_{2}$$

6 $$\frac{{{\text{dNS}}}_{1}}{{\text{d}}t}= {{\text{RSNS}}}_{2}-\mathrm{ vS}{{\text{NS}}}_{1}$$

7 $$\frac{{{\text{dNS}}}_{2}}{{\text{d}}t}={{\text{eSNS}}}_{1}-\mathrm{ \mu S}{{\text{NS}}}_{2}$$

Inducer juveniles (NI_1_) are produced at rate RI and removed from the system at a constant mortality rate (vI). Inducer mite reproduction benefits proportionally (b) from the presence of suppressors. Inducer reproduction is assumed to be density dependent following a Michaelis–Menten function, rI being the maximum reproduction rate of inducers, which is corrected for sex ratio, and *C* being the carrying capacity considering both phenotypes (*I* and *S*):

8 $${\text{RI}}={\text{rI}}\left({\text{max}}\left[0, 1-\frac{{{\text{NI}}}_{1}+{{\text{NI}}}_{2}+{{\text{NS}}}_{1}+{{\text{NS}}}_{2}}{{\text{C}}}\right]\right)$$

In the case of suppressor mites, suppressor juveniles (NS_1_) are produced at rate RS and removed from the system at a constant mortality rate (vS). As with inducers, suppressor reproduction is assumed to be density dependent following a Michaelis–Menten function, rS is the maximum reproduction rate of suppressors, corrected for sex ratio, and C carrying capacity considering both phenotypes (*I* and *S*):

9 $${\text{RS}}={\text{rS}}\left({\text{max}}\left[0, 1-\frac{{{\text{NI}}}_{1}+{{\text{NI}}}_{2}+{{\text{NS}}}_{1}+{{\text{NS}}}_{2}}{{\text{C}}}\right]\right)$$

Inducer juveniles (NI_1_) develop into inducer adults (NI_2_) with rate eI and suppressor juveniles (NS_1_) develop into suppressor adults (NS_2_) with rate eS. Adult inducers are removed from the system by a constant mortality rate µI while adult suppressors are removed by a constant mortality rate *µ*S.

#### Simulations

All simulations were performed over a spider mite development season of 98 days (Gotoh 1986; from June to September, which corresponds to 7 generations at 25 °C and 14 generations at 32 °C). In all cases, simulations were performed assuming a homogeneous environment in which strains of inducers and suppressors share the same plant, only with different initial densities of each. Initial densities were 90 inducers and 10 suppressors (individuals/plant), as suppressors are considered the rare phenotype in *T. urticae* (Blaazer et al. 2018). Note, the model did not allow for reproduction between strains. All simulations were conducted in R (R Core Team 2022) using the package deSolve (Soetaert et al. 2012).

#### Population dynamics modelled under two temperature regimes

Data from our experiments were used toparameterize the model and simulate four scenarios: (i) continuous mite development under 25 °C, (ii) continuous mite development under 32 °C, (iii) a heatwave of 32 °C for one week every month and (iv) a heatwave of 32 °C for two weeks every month. Model parameters for inducers and suppressors varied according to temperature (Table 1). For simulations (i) and (ii) temperature-relevant parameters were used. For simulations considering heat waves (i.e. iii and iv), the relevant parameters under the temperature at a specific point in time were used. In the case of modelling a one-week heat wave per month, at the start of the simulation parameter values relevant to 25 °C were used to simulate dynamics over three weeks followed by simulating one week with parameter values relevant to 32 °C. This cycle then repeated itself until the end of the simulation on day 98. For modelling a two-week heat wave per month, at the start of the simulation parameter values relevant to 25 °C were used to simulate dynamics over two weeks followed by simulating two weeks with parameter values relevant to 32 °C. As before, this cycle was repeated until the simulatio’'s end (day 98).

**Table 1:** Description of model parameters and values

Parameters	Description	Value	Units
	*Inducer biology at* 25 °C		
eI	Per capita developmental rate from egg to adult^a^	1/12.262	1/day
µI	Adult mortality rate^b^	0.062	/day
vI	Juvenile mortality rate^a^	0.309	/day
rI	Maximum rate of net reproduction^b^	8.599	Offspring/adult*day
	*Inducer biology at* 32 °C		
eI	Per capita developmental rate from egg to adult^a^	1/7.465	1/day
µI	Adult mortality rate^b^	0.159	/day
vI	Juvenile mortality rate^a^	0.414	/day
rI	Maximum rate of net reproduction^b^	12.263	Offspring/adult*day
	*Suppressor biology at* 25 °C		
eS	Per capita developmental rate from egg to adult^a^	1/11.857	1/day
µS	Adult mortality rate^b^	0.0419	/day
vI	Juvenile mortality rate^a^	0.261	/day
rS	Maximum rate of net reproduction^b^	8.186	Offspring/adult*day
	*Suppressor biology at* 32 °C		
eS	Per capita developmental rate from egg to adult^a^	1/7.429	1/day
µS	Adult mortality rate^b^	0.075	/day
vI	Juvenile mortality rate^a^	0.327	/day
rS	Maximum rate of net reproduction^b^	15.798	Offspring/adult*day
*C*	Carrying capacity	1000	Individuals
*b*	Increased benefit to inducers in presence of suppressors	1.25^c^	

^a^From empirical data obtained in this study from eggs developing on leaf discs; ^b^from empirical data obtained from adult females feeding on leaflets of whole plants, developing on leaf discs;^c^from empirical data obtained in Alba et al. 2015

## Results

### Effect of temperature on female survival, plant damage and oviposition

**Fig. 1:**
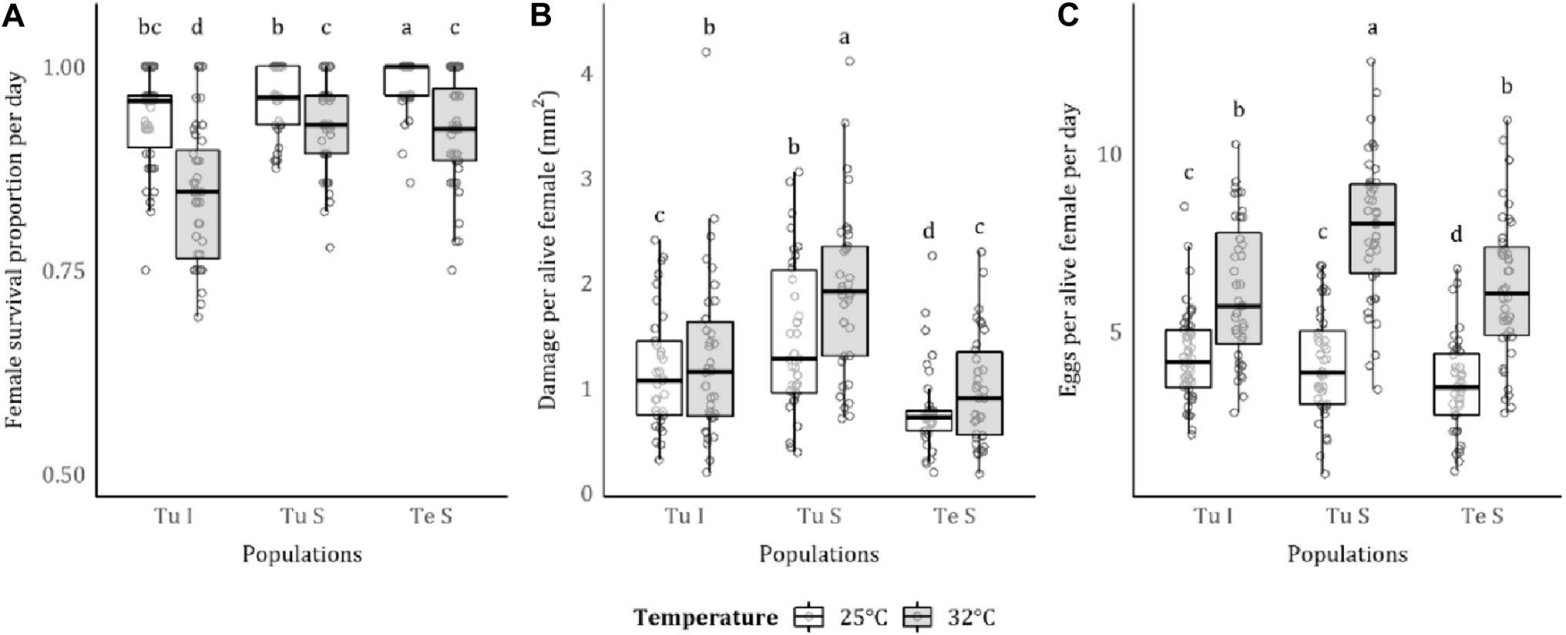
Effect of temperature on spider mite female survival. **A** Female survival proportion per day, **B** tomato leaflet damage area per alive female (mm^2^), and **C** eggs per alive female per day (i.e. fecundity). Each point denotes one biological replicate (leaflet, *n* = 40). White boxplots correspond to measures at 25 °C, and grey boxplots correspond to measures at 32 °C. Different lowercase letters indicate statistical differences, according to multiple comparison analyses performed using estimated marginal mean, in pairwise comparisons between all treatments. On the *x*-axis, “Tu I” denotes *T. urticae* inducer, “Tu S” denotes *T. urticae* suppressor, and “Te S” denotes *T. evansi* suppressor mites

The high temperature of 32 °C reduced survival for all mite strains, but the relative decline was different for each strain (mite*temperature: ${X}_{2}^{2}$ = 7.388, *p* = 0.025; mite*:* ${X}_{2}^{2}$= 10.664, *p* = 0.005; temperature: ${X}_{1}^{2}$ = 31.5573, *p* < 0.001; Fig. 1A). At 25 °C, inducer mites had the highest mortality, followed by *T. urticae* suppressor and *T. evansi* suppressor. At 32 °C, mortality was higher for the *T. urticae* inducer strain than for both suppressor strains.

Total feeding damage was corrected for the strain-specific mortality to calculate feeding damage per living female. There was a significant effect of temperature and strain on feeding damage (mite*temperature: ${X}_{2}^{2}$ = 1.636, *p* = 0.441; mite: ${X}_{2}^{2}$ = 72.045, *p* < 0.001; temperature: ${X}_{1}^{2}$ = 7.121, *p* = 0.008; Fig. 1B). Damage was higher at 32 °C than at 25 °C for all mite strains, with *T. urticae* suppressor causing the highest level of damage and *T. evansi* suppressor the lowest level at both temperatures.

Female fecundity was higher at 32 °C for all mite strains but the rank order of differences between strains changed across temperatures (mite*temperature: ${X}_{2}^{2}$ =10.069, *p* = 0.007; mite: ${X}_{2}^{2}$ = 10.435, *p* = 0.005; temperature: ${X}_{1}^{2}$ = 25.315, *p* < 0.001; Fig. 1C). At 25 °C, both *T. urticae* strains had higher fecundity per female than the *T. evansi* suppressor, but at 32 °C, *T. urticae* suppressor had higher fecundity.

### Effect of the higher temperature on *T. urticae* and *T. evansi* developmental time and survival

**Fig. 2:**
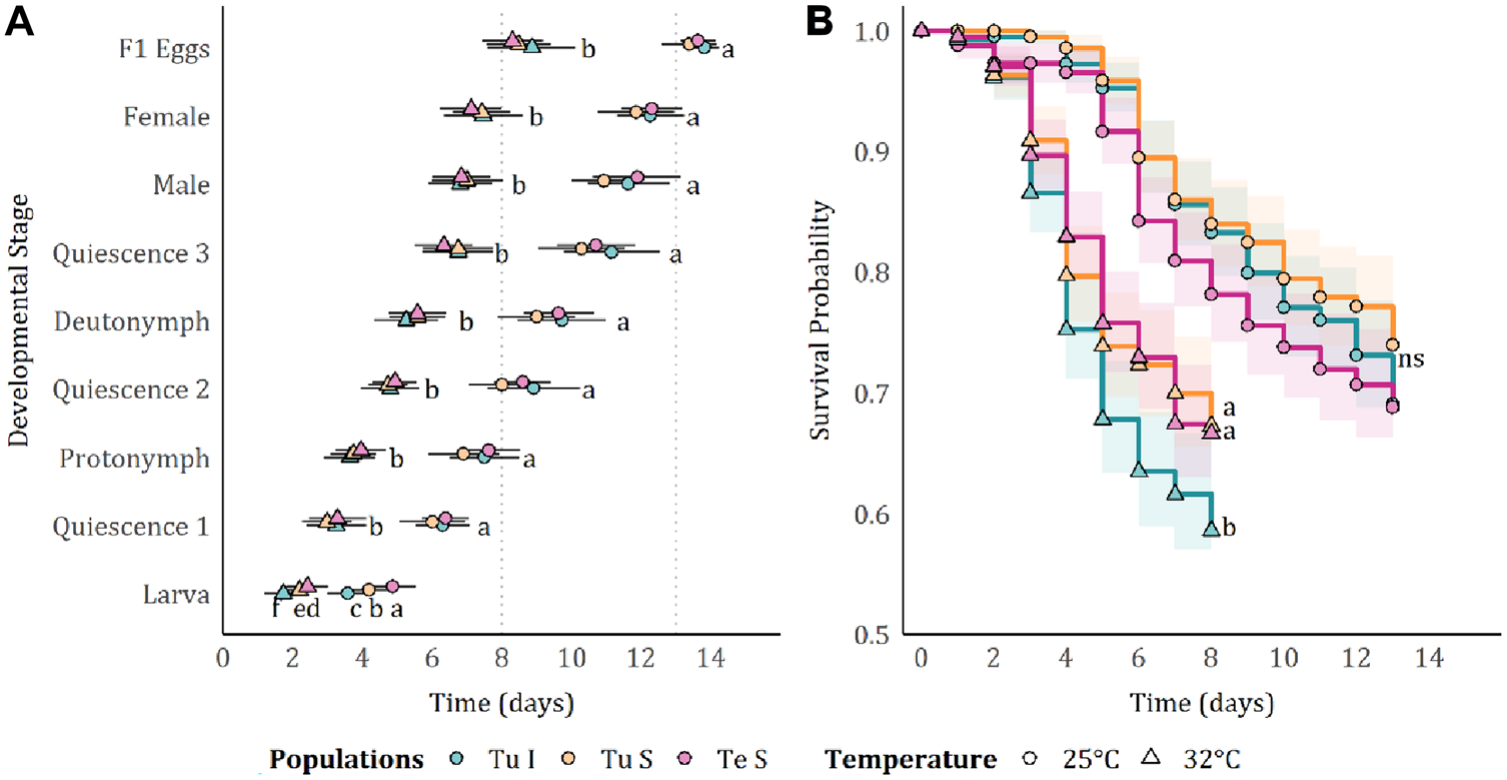
Effect of temperature on spider-mite development and survival. **A** Mean (± SD) number of days until each developmental stage. Dashed vertical lines mark the approximate number of days for a complete generation cycle (from egg to reproductive adult; at 32 and 25 °C; **B** mean (± 95% CI) survival probability for a complete generation cycle (from egg to reproductive adult). Different lowercase letters indicate statistical differences, according to multicomparison analysis performed using estimated marginal means, (**A**) in pairwise comparisons between all treatments within each developmental stage, and (**B**) in pairwise comparisons between all mite strains within 25 °C or 32 °C. ns codes for no significant differences; Different colours indicate a different treatment: blue—*T. urticae* inducer mites (“Tu I”), yellow—*T. urticae* suppressor mites (“Tu S”), and pink—*T. evansi* suppressor mites (“Te S”)

Egg-to-adult development was faster at 32 °C than at 25 °C for all three mite strains. The average developmental time (i.e. the time from egg stage to first oviposition according to Abou-Setta and Childers 1991) was reduced from, approximately, 13 days at 25 °C to, approximately, eight days at 32 °C (Table 1). The time to reach each next developmental stage was slower at 25 °C than at 32 °C for all mite strains (developmental stage*temperature: ${X}_{8}^{2}$ = 27.025, *p* < 0.001). Yet, this also differed across strains, though independent of temperature (developmental stage*mite: ${X}_{16}^{2}$ = 29.764, *p* = 0.019; developmental stage*mite*temperature: ${X}_{16}^{2}$ = 3.070, *p* = 1; Fig. 2A). Post hoc comparisons revealed that only the time needed for the egg to reach the larval stage differed across strains. For both temperatures, larvae appeared first in the *T. urticae* inducer, followed by *T. urticae* suppressor and lastly by the *T. evansi* suppressor.

When assessing the offspring survival probability at different temperatures, we observed that, at 25 °C, survival was similar between mite strains (mite: ${X}_{2}^{2}$ = 3.267, *p* = 0.195, Fig. 2B), while at 32 °C, survival was different between strains (mite: ${X}_{2}^{2}$ = 12.932, *p* = 0.002, Fig. 2B). At 32 °C, survival of the inducer *T. urticae* was lower than that of suppressor mites (both *T. urticae* and *T. evansi*).

We observed that the effect of temperature on the proportion of female offspring is different for the mite strains (mite*temperature: ${X}_{2}^{2}$ = 7.299, *p* = 0.026, mite: ${{\text{X}}}_{2}^{2}$ = 0.8540, *p* = 0.652, temperature: ${X}_{1}^{2}$ = 5.500, *p* = 0.019, Figure S1). Although at 25 °C all strains have a similar proportion of females, at 32 °C the *T. evansi* suppressor strain had a higher proportion of female offspring than the *T. urticae* inducer strain, with the *T. urticae* suppressor strain having an intermediate proportion of female offspring.

### Effect of temperature on transcript accumulation of *effector 84* and several plant defence genes

**Fig. 3:**
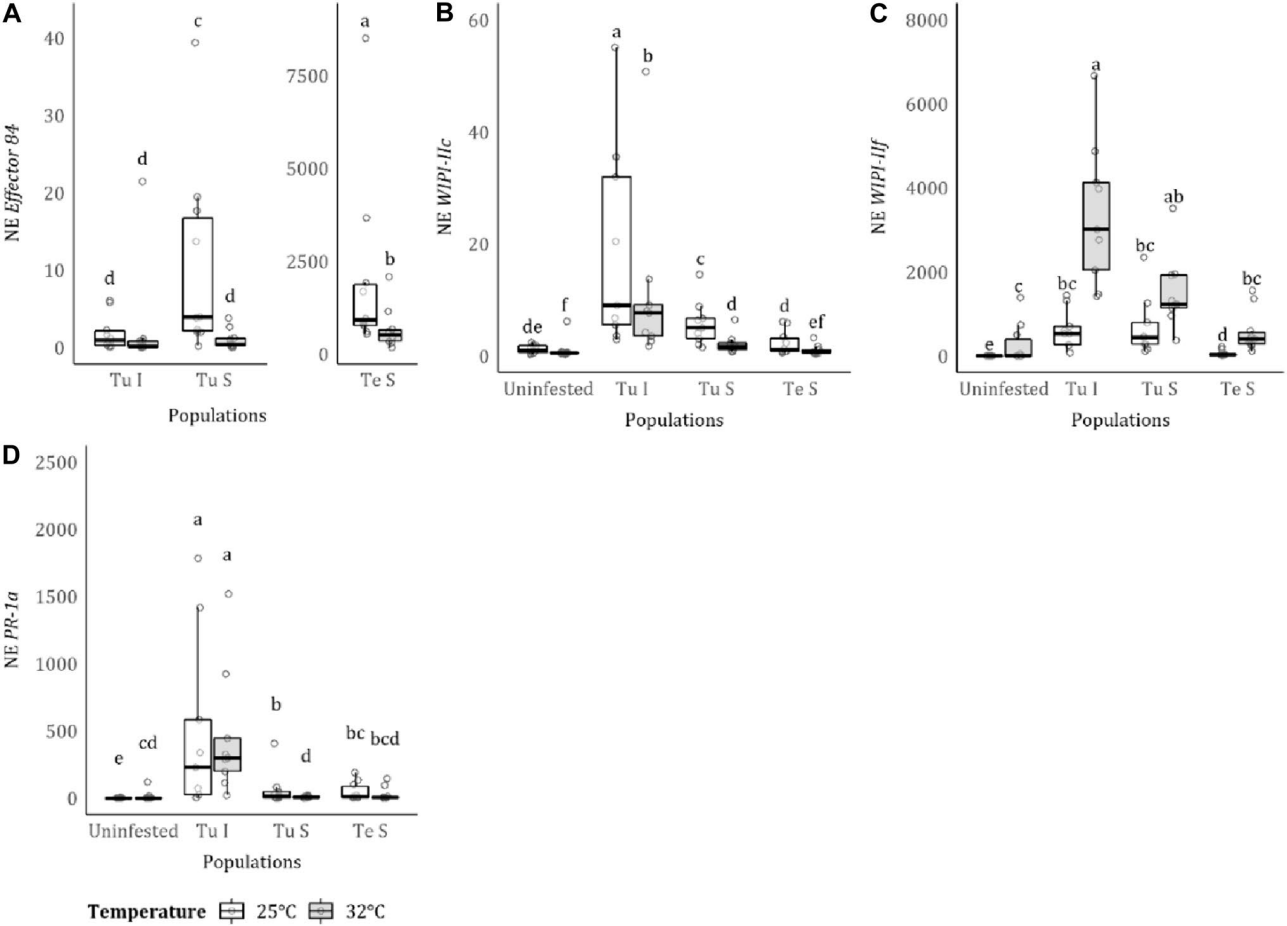
Effect of temperature on the normalized expression (NE) of **A** *effector 84*; on JA defense markers **B** *WIPI-IIc* and **C** *WIIPI-IIf*, and on SA defense marker **D** *PR-1a*. Each point denotes one biological replicate (leaflet, *n* = 20). White boxplots correspond to measures at 25 °C, and grey boxplots to measures at 32 °C. Different lowercase letters indicate statistical differences, according to multicomparison analysis performed using estimated marginal mean, in pairwise comparisons between all treatments. On the *x*-axis, “Tu I” denotes for *T. urticae* inducer, “Tu S” denotes for *T. urticae* suppressor, and “Te S” denotes for *T. evansi* suppressor mites

For the *effector 84* transcript accumulation, the interaction between mite strain and temperature was significant (mite*temperature: ${X}_{2}^{2}$ =12.871, *p* = 0.002; mite: ${X}_{2}^{2}$ = 187.087, *p* < 0.001; *temperature:* ${X}_{1}^{2}$=0.519, *p* = 0.471; Fig. 3A). This was mainly explained by a decrease in effector transcript accumulation in the suppressor strains at 32 °C, relative to 25 °C, which was not observed in the inducer strain. For the *T. urticae* suppressor, transcript levels at 32 °C decreased to levels similar to that of the inducer (a tenfold decrease on average) compared to 25 °C, while for the *T. evansi* suppressor levels decreased approximately threefold at 32 °C relative to 25 °C.

Plant defence marker gene nEs differed between temperatures and mite strains. For the JA marker *WIPI-iIc,* there was an overall decrease in expression at higher temperature (mite*temperature: ${X}_{3}^{2}$ = 3.810, *p* = 0.283; mite: ${X}_{3}^{2}$ = 109.042, *p* < 0.001; temperature: ${X}_{1}^{2}$ = 10.642, *p* < 0.001, Fig. 3B). *WIPI-iIc* expression was higher in leaves infested with the *T. urticae* inducer, followed by leaves infested with *T. urticae* suppressor and those with *T. evansi* suppressor accumulating levels similar to the uninfested plants. This pattern was similar at both temperatures. For the JA marker *WIPI-iIf* the interaction between mite strain and temperature was significant (mite*temperature: ${X}_{3}^{2}$ = 45.971, *p* < 0.001; mite*:* ${X}_{3}^{2}$ = 195.229, *p* < 0.001; temperature: ${X}_{1}^{2}$ = 117.480, *p* < 0.001, Fig. 3C). *WIPI-iIf* expression in plants infested with *T. urticae* was higher than in uninfested plants at both temperatures and expression was especially elevated upon infestation with *T. urticae* inducer at 32 °C. For plants infested with the *T. evansi* suppressor, *WIPI-iIf* transcript levels were similar to uninfested plants at both temperatures.

The interaction between temperature and mite strain significanty affected the expression of the SA marker gene *PR-1a* (mite*temperature: ${X}_{3}^{2}$ = 19.635, *p* < 0.001; mite: ${X}_{3}^{2}$ = 66.942, *p* < 0.001; *temperature:* ${X}_{1}^{2}$ = 11.758, *p* < 0.001, Fig. 3D). Similar to *WIPI-iIf*, uninfested plants showed a higher NE at 32 °C compared to the expression at 25 °C. Plants infested with the *T. urticae* inducer strain accumulated the highest levels of *PR-1a* transcript, with no differences between temperatures. Plants infested with either suppressor strains accumulated lower *PR-1a* transcript levels than plants infested with *T. urticae* inducer at both temperatures.

### Effect of temperature on the population dynamics of inducer and suppressor strains of *T. urticae*

**Fig. 4:**
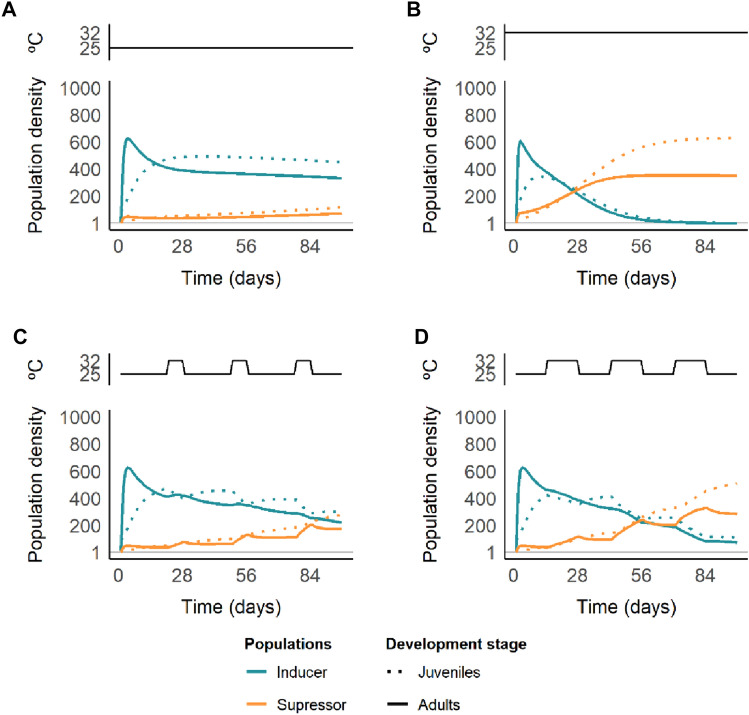
Strain density and dynamics of inducer (blue) and suppressor (orange) strains of *T. urticae* at a constant temperature of **A** 25 °C, **B** 32 °C, and in a heatwave scenario in which **C** three weeks of 25 °C is followed by one week of 32 °C, and **D** two weeks of 25 °C is followed by two weeks of 32 °C for 98 days. In each panel, the top graph represents the temperature fluctuations through time. The *y*-axis indicates the number of individuals (doted lines—juveniles; solid lines—adults). The grey line intercepting the y-axis at 1 represents the minimal number of individuals for a strain to be alive

From the two-mite phenotype model, considering inducer and suppressor strains of *T. urticae*, we observed that, at a constant temperature of 25 °C, the density of suppressor mites is maintained lower than that of inducer mites, with a progressive increase until the end of a mite season (i.e. 98 days, or approximately, three months June to September, Gotoh 1986, Fig. 4A). However, at a constant temperature of 32 °C, although the initial peak of inducer mites, after approximately 28 days, suppressor mites become more common in the population (Fig. 4B). At this temperature, inducer mites do not persist until the end of the season.

In the 1 week heat wave per month scenario (Fig. 4C), we observed a similar pattern to the one for a constant temperature of 25 °C. However, the increase in suppressor mite density is more rapid and reaches values similar to those of the inducing strain at the end of the season. In the two weeks per month heatwave regime (Fig. 4D), although we observed a similar pattern to the one for a constant temperature of 32 °C with suppressor mites surpassing inducer mites, the shift occurs later (i.e. ~ day 56, after two months). At the end of the season, inducer mites do persist, but at a very low density.

## Discussion

Here we present data that suggest that mites capable of suppressing plant defences may outcompete the inducer mites at 32 °C but not at 25 °C. At 32 °C, all mite strains caused more feeding damage to their tomato host, especially for *T. urticae* suppressor strain. This occurred simultaneously with a reduced expression of *effector 84* for both suppressor mite strains while the pattern of induction/suppression did not change significantly with temperature. Mite fecundity was higher for all strains at 32 °C, yet, at this temperature, juvenile and adult survival of *T. urticae* inducer mites was lower than that of the suppressor strains.

### Plant damage and fecundity increased from 25 to 32 °C, but mite survival decreased

Plant damage per female was higher at 32 °C than at 25 °C, showing an increase in mite voracity (Fig. 1B). This could be explained by an acceleration of metabolism and the life cycle at high temperatures, therefore, reflecting feeding damage (Deutsch et al. 2018). Indeed, this was also observed in several other arthropod species (e.g. increased feeding in *Popillia japonica*, Lemoine et al. 2013; Deutsch et al. 2018).

We also observed that the fecundity of all mite strains was higher at 32 °C than at 25 °C (Fig. 1C). However, at the same time, offspring mortality (Fig. 2B) and adult mortality (Fig. 1A) were also higher at 32 °C, especially in the case of the inducer *T. urticae* strain. The observed increased fecundity may be a compensatory strategy to overcome the effects of higher temperature on mortality. Fecundity compensation is found in many species in response to different stresses, such as exposure to pesticides (e.g. in aphids, Ayyanath et al. 2013), parasite infection (e.g. in snails, Blair and Webster et al. 2007), predation (e.g. in fish, Grégoir et al. 2018a, b) and also temperature (e.g. aphids, Ma et al. 2015). Alternatively, it might be a consequence of a reduction in the generation time of mites at higher temperature. Indeed, it has also been shown for *T. urticae* that fecundity is inversely proportional to developmental rate (Flexner et al. 1989). We found that mite development was reduced from approximately 13–8 days at 32 °C (Fig. 2A), which is in line with previous studies in these species (*T. urticae:* Riahi et al. 2013; *T. evansi:* Bonato 1999). Although not fully understood, it probably reflects an accelerated life cycle.

### Suppression of plant defences is not temperature-dependent, despite reduced expression of *effector 84* at 32 °C

We hypothesized that the expression of *effector 84* would increase with feeding (assuming an increase in saliva production) which would be reflected in increased feeding damage. However, we observed a decrease in effector expression at 32 °C in both suppressor strains while in the *T. urticae* inducer strain (whose *effector 84* NE was relatively low compared to the *T. urticae* suppressor) the decrease was not significant (Fig. 3A). This decrease in the expression of *effector 84* was not accompanied by an increase in the expression of plant defence genes as plants infected by suppressor mites still had lower expression of immune genes at 32 °C (Fig. 3B–D). That could indicate a more efficient defence suppression.

Previously, it was reported that the expression of *effector 84* is plastic and responds readily to the presence of competitors, varies across life stages and sexes and follows the diurnal cycle (Liu et al. 2020a, b; Schimmel et al. 2017). In a study by Liu et al. (2020a), the authors found that in the dark, *effector* 84 transcript levels were lower than under light for both *T. urticae* inducer and *T. evansi* suppressor strains, although differences were larger for the *T. evansi* suppressor (Liu et al. 2020a). However, the lower *effector 84* expression at 32 °C was only significant for both suppressor strains but not for the *T. urticae* inducer. This observation, together with the results of Liu et al. 2020a, indicates that the plasticity of *effector 84* expression has a much wider dynamic range in suppressor mites than in the inducers and not only in *T. evansi,* as reported previously (Schimmel et al. 2017), but also in *T. urticae*. Interestingly, inducer and suppressor *T. urticae* produce distinct *effector 84* proteins that group into different clades (Teodoro-Paulo et al. 2023). The data in the present study indicate that not only coding sequences may differ, as showed in Teodoro-Paulo et al. 2023, but also their regulatory sequences or their regulators (e.g. transcript factors), suggesting that this effector plays a more prominent role in suppressors than in inducer mites, as it is more plastic for suppressors. Other effectors, like GOX in Helicoverpa zea, are affected by temperature. The activity of the GOX enzyme was significantly reduced at 35 °C; however, this was not accompanied by significant changes in the expression of GOX (Paudel et al. 2020) as observed for *effector 84*. This highlights the need for a better understanding of the functions of the different *effector 84* paralogs, their tomato target(s) and other salivary components that could interfere with plant defences in order to predict whether or not this gene may be a target of natural selection in strains and drive adaptation of *T. urticae* strains to novel hosts upon exposure to rising temperatures.

At 32 °C, uninfested plants had lower levels of *WIPI-IIc* (Fig. 3B) and higher levels of *WIPI-IIf* (Fig. 3C) and *PR*-*1a* (Fig. 3D) than at 25 °C, indicating tomato defence gene expression is directly influenced by temperature and not only mite infestation. A temperature of 32 °C is in the range of optimal temperatures for growing tomato plants (Hochmuth and Hochmuth 2012) and, in crop fields, fluctuations in temperature that include 32 °C are frequent during the growing season (Zhu et al. 2021). We found minimal differences in plant development at the two temperatures, with plants being slightly taller at 32 °C, but with approximately the same number of leaves and leaflet area (see supplementary materials Figure S2). Although at 32 °C the constitutive expression of two of the three markers genes was higher, plants do not seem more resistant to the mites since they caused more damage and had higher fecundity at this temperature.

### Higher temperatures may favour mites that can suppress defences over inducers

Modelling the dynamics of the *T. urticae* inducers and suppressors predicts that the high abundance of inducers at 25 °C diminished at 32 °C resulting in a complete displacement of inducer mites at the end of the season (Fig. 4A, B). This could be attributed mainly to the differences in mortality, with that of suppressor mites being lower than that of inducers at 32 °C. The models predict that in a field population composed of a mixture of inducer and suppressor mites, suppressor mites will overtake the population on tomato plants at 32 °C (Fig. 4B). Rapid temperature-guided selection of suppressors in the laboratory also may help to understand why defence suppression persists within natural strains even though the suppressed plant is a common good that also benefits inducer competitors (Sarmento et al. 2011; Alba et al. 2015; Blaazer et al. 2018). Indeed, the reproductive benefit conferred by the suppressor mites to the inducer mites was not enough for inducers to be maintained in the population in our model.

With one-week heat waves per month (Fig. 4C), our model predicts that near the end of the season, the ratio between suppressor and inducer mites will have increased such that suppressor mites have become more common during the season. Having two heat waves per month (Fig. 4D), our model predicts an almost complete displacement of inducer mites by suppressor mites at the end of the season. We acknowledge that our models are parametrized simplistically in the sense that in the field or greenhouse, temperatures vary less drastically than during our simulated heat waves. However, we consider them to be a strong conceptual starting point for predicting trends that will emerge more often and more strongly due to climate change. Indeed, with the increase in extreme weather events, such as heat waves, and longer summers (Lhotka and Kyselý 2022), it seems evident that suppressor mites will have a selective advantage allowing spider mites to adapt faster to novel hosts. This could exacerbate the already predicted increase in yield loss (10–25% per °C warming), which does not account for pests, which will be especially noticeable in temperate zones (Deutsch et al. 2018).

### Conclusion and perspectives

Our results show that induction and suppression of plant defences by spider mites are not influenced by the high temperature tested here, however, life history traits of mites are significantly affected. Also, we advance the knowledge on the *effector 84* plasticity, with our results suggesting that this effector has a more prominent role for suppressor rather than inducer mites. Based on the results of the population models, we advocate that studying how temperatures influence pest adaptation, for instance in Europe, is of extreme importance for adjusting pest management and crop production policies, especially now that climate change is accelerating.

## Supplementary Information

Below is the link to the electronic supplementary material.

Supplementary file1 (DOCX 487 KB)
