# Supplementary material for: Rising temperatures favour defence-suppressing herbivores
Source: J Pest Sci (2004). 2024 Apr 20;98(1):535–48. doi: 10.1007/s10340-024-01781-2 (PMC11923023; doi:10.1007/s10340-024-01781-2)
Supplement: Supplementary file 1 — Supplementary file1 (DOCX 487 KB) [file 10340_2024_1781_MOESM1_ESM.docx]

Rising temperatures favour defence-suppressing herbivores

Jéssica Teodoro-Paulo^1,2,3^ (ORCID: 0000-0003-2933-9940), Jacques A. Deere^2^ (ORCID: 0000-0001-6736-2223), João Valeriano-Santos, Steven Charlesworth^1^, Alison B. Duncan^3^ (ORCID: 0000-0002-6499-2913) Merijn R. Kant^2^(ORCID: 0000-0003-2524-8195), Juan M. Alba^2,4^ (ORCID: 0000-0003-4822-9827) ^1^cE3c - Centre for Ecology, Evolution and Environmental Changes & CHANGE - Global Change and Sustainability Institute, Faculty of Sciences, University of Lisbon, Lisbon, Portugal

^2^Institute for Biodiversity and Ecosystem Dynamics, University of Amsterdam, Amsterdam, The Netherlands

^3^Institut des Sciences de l’Évolution, University of Montpellier, CNRS, IRD Montpellier, France

^4^Institute for Mediterranean and Subtropical Horticulture La Mayora (IHSM), CSIC-UMA, Málaga, Spain

Supplementary materials

Table S1 **Experimental design with infestation scheme and distribution of the experiments and replicates through temporal blocks.**

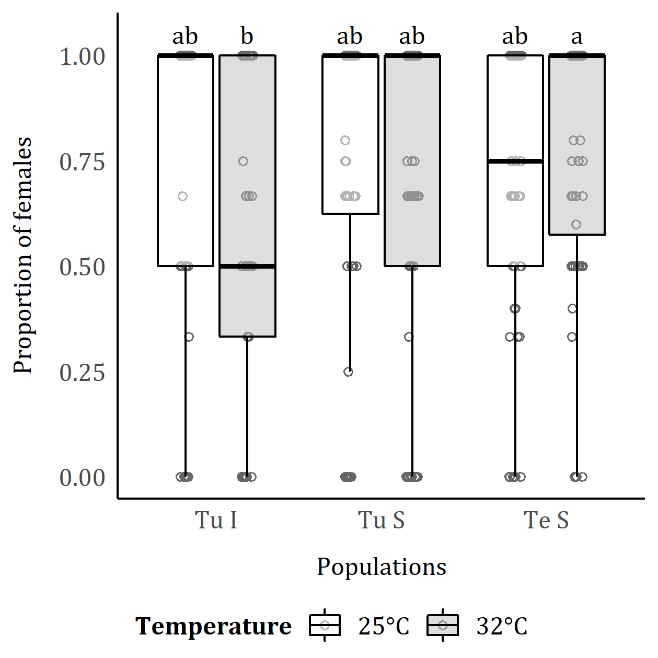


Figure S1. **Effect of high temperatures on proportion of adult female offspring.** Each point denotes one replicate (i.e., leaf disc). White boxplots code for 25 °C and grey boxplots for 32 °C. Different lowercase letters indicate statistical differences, according to multicomparison analysis performed using estimated marginal mean, in pairwise comparisons between all treatments.

**Effect of high temperatures on plant growth**

To evaluate if plant development is affected when exposed to high temperatures for 3 days, we assessed plant height (in cm), the number of new leaves and the total leaflet area on uninfested and infested plants at the end of the assay, at both temperatures. This was done for ten plants infested with each mite strain and ten clean plants, for both temperatures, from blocks 5 to 9 (n = 10).

Since normality was met (Shapiro-Wilk test: p = 0.280), and the variances were homogeneous (Levene’s test: p = 0.933), a generalized linear mixed model (GLMM) with a normal error structure (lmer, lme4 package, Bates *et al.* 2015) was used to determine the effect of temperature and mite strain on plant height (cm). A GLMM assuming a Poisson distribution (lmer, lme4 package, Bates *et al.* 2015) was used to evaluate the effect of temperature and mite strain on the number of leaves. To evaluate differences in total leaflet area as a proxy for leaf expansion, a GLMM model assuming a Gamma distribution and a log effect of the fixed factors (family = Gamma(link=”log”), lmer, lme4 package, Bates *et al.* 2015). All models included *mite* (*T. urticae* inducer, *T. urticae* suppressor, *T. evansi* suppressor or uninfested plants), *temperature* (25 °C or 32 °C) and their interaction (*mite:temperature*) as fixed explanatory variables, and *block* as a random variable.

When significant differences were found, multiple comparisons were performed using estimated marginal means (emmeans, emmeans package) (Lenth *et al.* 2019) and the p-values corrected using the false discovery rate (FDR) method (α= 0.05) (Benjamini & Hochberg, 1995).

Plants exposed to 32 °C were marginally, but significantly taller than plants maintained at 25 °C, with no effect of the mite strain infesting the plant (*mite:temperature*: $X_{3}^{2}$ = 0.084, p = 0.994; *mite:* $X_{3}^{2}$ = 3.920, p = 0.270; *temperature:* $X_{1}^{2}$ = 3.991, p = 0.046; Figure S2A). The number of leaves was not significantly affected by either temperature, mite strain or their interaction (*mite:temperature*: $X_{3}^{2}$ = 0.340, p = 0.952, *mite:* $X_{3}^{2}$ = 0.742, p = 0.863; *temperature:* $X_{1}^{2}$ = 0.285, p = 0.604; Figure S2B). The same was observed for total leaf area (i.e., leaf expansion; *mite:temperature*: $X_{3}^{2}$ = 0719, p = 0.869, *mite:* $X_{3}^{2}$ = 1.294, p = 0.731; *temperature:* $X_{1}^{2}$ = 0.090, p = 0.764; Figure S2C).


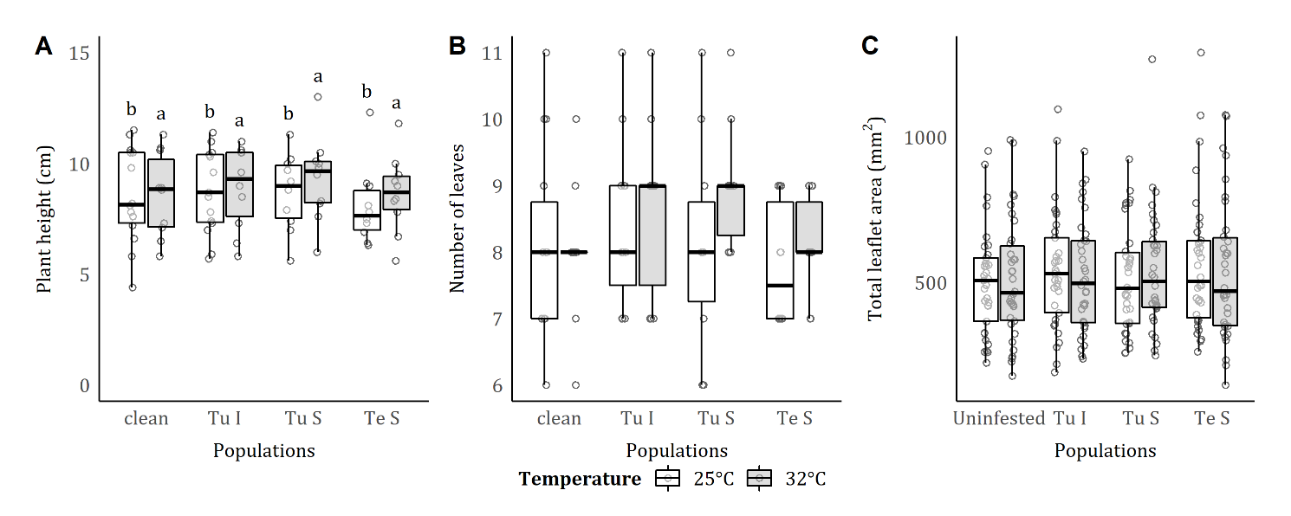
Figure S2. **Effect of high temperatures on plant growth.** (A) Plant hight (cm), (B) Number of leaves and (C) Total leaflet area (mm^2^), Each point denotes one biological replicate. White boxplots correspond to measures at 25 °C and grey boxplots to measures at 32 °C. Different lowercase letters indicate statistical differences, according to multicomparison analysis performed using estimated marginal mean, in pairwise comparisons between all treatments. On the x-axis, “Tu I” denotes for *T. urticae* inducer, “Tu S” denotes for *T. urticae* suppressor, and “Te S” denotes for *T. evansi* suppressor mites.
